# An Update of Research Animal Models of Inflammatory Bowel Disease

**DOI:** 10.1155/2021/7479540

**Published:** 2021-12-13

**Authors:** Zineb Baydi, Youness Limami, Loubna Khalki, Nabil Zaid, Abdallah Naya, El Mostafa Mtairag, Mounia Oudghiri, Younes Zaid

**Affiliations:** ^1^Immunology and Biodiversity Laboratory, Department of Biology, Faculty of Sciences Ain Chock, Hassan II University, Casablanca, Morocco; ^2^Laboratory of Health Sciences and Technologies, Higher Institute of Health Sciences, Hassan First University of Settat, Settat, Morocco; ^3^Mohammed VI University of Health Sciences (UM6SS), Casablanca, Morocco; ^4^Biology Department, Faculty of Sciences, Mohammed V University, Rabat, Morocco; ^5^Research Center of Abulcasis University of Health Sciences, Rabat, Morocco

## Abstract

Inflammatory bowel disease (IBD) is a group of chronic disorders that includes two main disease forms, Crohn's disease, and ulcerative colitis. The understanding of the intestinal inflammation occurring in IBD has been immeasurably advanced by the development of the now numerous murine models of intestinal inflammation. The usefulness of this research tool in IBD arises from a convergence of underlying genetic susceptibility, immune system dysfunction, environmental factors, and shifts in gut microbiota. Due to the multifactorial feature of these diseases, different animal models have been used to investigate the underlying mechanisms and develop potential therapeutic strategies. The results of preclinical efficacy studies often inform the progression of therapeutic strategies. This review describes the distinct feature and limitations of each murine IBD model and discusses the previous and current lessons from the IBD models.

## 1. Introduction

Inflammatory bowel disease (IBD) is a multifactorial or even multigenic disease, affecting roughly 1 million to 1.5 million patients in the United States [1–3]. The population of North Africa and Europe are the most affected by IBD; however, the epidemiology of this disease increased considerably in the mid-twentieth century [4].

In 1957, Dr. Kirsner and colleagues first invented an experimental colitis model, which was induced in rabbits by sensitization to crystalline egg albumin together with small rectal instillation of dilute formalin [5]. They tested this model by sensitizing rabbits to crystalline egg albumin by a rectal instillation technique of dilute formalin. Different types of chemically induced colitis models, primarily in rats, have since been developed [6]. In 1981, a new animal model was discovered, the cotton-topped tamarind, living in a small geographic area in Colombia was identified in order to induce spontaneous colitis.

In 1990, an adoptive T-cell transfer system to induce colitis in immunodeficient mice has been developed by Dr. Powrie and his colleagues [7]. This model system has provided a significant contribution to the development of the new concept on “regulatory T-cells” [8]. In the same year, they have identified genetically modified rats carrying the human HLA-B27 gene to develop colitis [9]. A major turning point in IBD research was then made in 1993 by the discovery of spontaneous colitis in three different kinds of knockout (KO) mice: interleukin (IL)-2 KO8402910, IL-10 KO, and T-cell receptor (TCR) KO mice [10]. Indeed, there have since been well over 40 different kinds of genetically engineered KO mouse strains and congenital gene mutant mouse strains found to develop colitis and/or ileitis spontaneously.

These experimental models of IBD have provided important contributions not only for understanding the basic mechanism of IBD but also for developing important therapeutic interventions against IBD [11–13]. Genetically modified mice clearly suggest the spontaneous development of colitis/ileitis in many alternative styles of strains whose IBD is mediated by extremely complicated mechanisms [12]. They identified more than 71 susceptibility genes in CD and 47 susceptibility genes in UC; these results can really reinforce the experimental model of IBD [14, 15]. It is approved that genetically modified mice lacking sensitivity (KO) or overexpressing (transgenic (Tg)) to IBD or candidate genes spontaneously develop intestinal inflammation. They include IL-10 KO, STAT3 KO, XBP1 KO, IL-2Ra KO, TNFSF15 Tg, and IL-7 Tg mice. This review summarizes the IBD mouse models currently available, in addition to the limitations for each model.

## 2. Chemically Induced Experimental Models

### 2.1. DSS-Induced Colitis Model

DSS is a highly water-soluble compound and gives a clear solution when dissolved in water. The colitogenic potential of DSS depends on its relative molecular mass [16].

DSS-induced inflammation occurred in the lamina propria of these mice but only in the areas underlying the defective epithelium, assuming that entry of commensal microorganisms into a normally normal lamina propria can induce an inflammatory response. A similar phenomenon occurs after administration of sodium dextran sulfate (colitis DSS); a sulfated polysaccharide that is toxic to the colonic epithelium causes damage to epithelial cells and an immune response that changes the mucosal barrier function throughout the colic epithelium. Thus, the administration of DSS to the mice is done in their drinking water for a short period leads to the induction of a highly reproducible acute inflammation properly limited to the colon and characterized by erosions/ulcers, loss of crypts, and infiltration of granulocytes [17].

In general, 36 to 50 kDa is the recommended weight during the induction of colitis. A lower molecular weight of DSS (5 kDa) can cause milder colitis, while a higher and higher DSS (500 kDa) does not cause colonic damage. As long as the DSS powder is stable at room temperature, it is preferable to prepare DSS water on the day of administration. DSS water should be mixed with a magnetic stir bar and totally dissolved before administration. Undissolved salt can block the outlet of a bottle or otherwise influences the water intake, which can potentially cause erroneous results. It is recommended to frequently check the turbidity inside the bottle as the turbidity indicates microbial growth and must be replaced with a new bottle containing freshly prepared DSS water. This is very essential, especially when inducing chronic colitis with several alternating DSS-water cycles and when preparing DSS solutions; the same water should be given to the control groups [16].

Experimentally, on the first day of DSS administration (day 0), the protocol is carried out according to several successive steps: control and exposed mice must be labeled (using the ear punch or any other practical method) should be weighed, and preferably, the average group weight should be balanced to eliminate any significant weight differences between groups. The labeling of mice makes it possible to follow their sensitivity to the pathology induced by DSS, as well as the weight makes it possible to follow the success of the protocol. It would be better to minimize the mixing of mice over 4 weeks old between different cages, especially males, as they can injure themselves by fighting. It is necessary to collect stool before administration of DSS, as they can be used as a control when assaying fecal markers of inflammation. If you are working with a compound for its anti-colitogenic properties, determine the route, dose, volume, vehicle, and method (prophylactic or therapeutic) of administration. The volume administered is 0.2 ml. The DSS-water solution is prepared by mixing an appropriate concentration of DSS in distilled water by weighing the DSS powder and mixing until a clear solution is obtained. Fill the cage water bottle with 100 ml DSS water, enough for 5 mice for 2-3 days. Control mice should obtain the same amount of water without DSS in the same period [16].

During the first three days, body weight may increase slightly and start to decrease gradually with the onset of bleeding. There is no standard and quick rule that the DSS should be administered for 7 days; the investigator can determine when the mice should be sacrificed based on significant loss of body weight and bloody diarrhea. As an example, mice deficient in MyD88 have pathology induced by DSS much earlier than wild-type mice [16].

### 2.2. TNBS-Induced Colitis Model

TNBS is a small haptenizing molecule, which is not antigenic, but when it binds to a host protein, it induces an immune response. The administration of TNBS is considered to be a preclinical mouse model inducing Crohn's disease (CD). This model generates an immune response mediated by Th1, characterized by an infiltration of CD4 T-cells, neutrophils, and macrophages. The result of this inflammatory response produces transmural colitis.

TNBS colitis has been used extensively for the study of immunological aspects, since it is a model that resembles features of Crohn's disease, counting cytokine secretion profiles, oral tolerance mechanisms, and the effects of potential immunotherapy.

Experimentally, TNBS is administered intrarectally to rodents after its dissolution in alcohol in order to cause colitis. Alcohol is not only considered as solvent or vehicle solution in biochemical reactions but also helps produce intestinal inflammation by breaking the mucosal barrier [18, 19]. Many researchers have demonstrated that anesthesia should be preceded by a fast 12 to 24 hours before induction using ether/halothane/chloral hydrate and so on. The most suitable method for the induction of colitis in rats involves the administration of 10 mg TNBS mixed with 0.25 ml of 50% ethanol and, then, instillation in male/female Wistar rats using a medical-grade polyurethane catheter (external diameter 2 mm) for internal feeding approximately 8 cm proximal to the anal border. After instilling the hapten, the head-down position is maintained so as to forestall leaks and distribute the hapten uniformly for 1 to 3 minutes. After 2 to 6 days of colitis induction, the rats are sacrificed by decapitation in order to assess the degree of inflammation of the colon according to different histological and immunohistochemical techniques [20–22]. The protocol can be modified by varying the dose and the volume of the TNBS solution without forgetting the change in the alcohol concentration in order to induce IBD to different degrees in Sprague Dawley or Wistar rats of both sexes.

The concentration of TNBS administered varies according to the recommended protocol and according to the investigator; some of the researchers use 0.8 ml of 5% TNBS in 50% (v/v) ethanol [23], and there are others who use 1 ml of 3% TNBS in 40% ethanol [24] and 0.5 ml of TNBS (100 mg/kg) [25]. Qin et al. compared the influence of variation in TNBS dose, ethanol concentration, and depth of the positioning of instillation in TNBS that induces PI-IBS. TNBS administered to rats at different doses (5, 10, and 20 mg/0.8 ml per rat) gave a sign that TNBS induces acute inflammation and damage at a dose dependent on high and median doses being more effective. It was found that the pathological score, that is, inflammation and visceral hyperalgesia was more significant in TNBS-50% ethanol-treated rats. However, it was demonstrated that instillation of a 5 mg/kg dose of TNBS at a depth of 8 cm/4 cm generates a similar severity of colonic inflammation [26]. Yang et al. demonstrated that recurrent ulcerative colitis model may also be induced in Wistar rats by instilling TNBS (100 mg/kg) into the colon through an obtuse cannula [27].

A strain of mouse SJL/J mainly represented TNBS colitis in mice, which is very sensitive to the induction of colitis. In point of fact, many other strains of mice are also frequently used for the development of colitis, especially BALB/C, C57BL/6, and so on. Overall, this involves low-dose rectal application (100 µl of 0.5 mg TNBS in 50% ethanol) for the induction of colitis that will lead to chronic transmural colitis with severe diarrhea, loss of weight, and rectal prolapse, a disease that mimics certain features of Crohn's disease in humans [18]. Moreover, TNBS also induces significant changes in the morphology, mechanical properties, and pharmacological response of circular muscle layer of the distal colon as compared to proximal counterpart in mice to mimic human ulcerative colitis [28]. The typical method adopted for inducing colitis in C57BL/6 mice involves intrarectal administration of 200 mg/kg TNBS dissolved in 30% ethanol via a catheter approximately 3∼4 cm proximal to anus. After 3 days, mice are sacrificed to carry out a histological examination of colon tissues [29]. Another researcher reported that colitis can be induced by instilling 20 mg of TNBS dissolved in 0.4 ml of 50% ethanol aqueous solution into the colon lumen [28]. TNBS results in infiltration of inflammatory cells within 2 hrs after administration, but typical signs of chronic inflammation develop after 48 hrs [28].

The exact mechanisms responsible for TNBS-induced IBD are poorly understood. Various scientists proposed different mechanisms for explaining the pathophysiological features of TNBS-induced IBD. It is reported that L-type Ca^2+^ channel currents are downregulated, whereas adenosine triphosphate (ATP) sensitive K+ channels are upregulated in gastrointestinal smooth muscle cells after administering TNBS to mice, which induces hyperpolarization of the gastrointestinal smooth muscle cells and thus results in reduced colonic contractility [30].

### 2.3. Oxazolone-Induced Colitis Model

Oxazolone is a haptenizing agent widely used to induce colitis in mice in order to evaluate the pathological processes involved in the perpetuation of ulcerative colitis. This model of colitis generates an immune response mediated by Th2 cells [31]. In addition, it has been shown that colitis induced by oxazolone resembles human ulcerative colitis on the basis of manifestations of inflammation of the mucous membranes, epithelial microulcerations, and histopathological changes in the distal colon [31, 32]. Different strains of mice were used to observe immunological responses, including C57BL/6J, BALB/CJ, and SJL/J. The C57BL/6J (C57/BL6 or C57/BL10) and SJL/J strains tend to have a Th1-mediated immune response, while BALB/J have a strong inclination towards a Th2 phenotype immune response [33]. Mice of the C57 strain resist the induction of colitis with oxazolone; therefore, to achieve this induction of colitis in the C57 strains, it is necessary to proceed first by a presensitization treatment. The mouse strain SJL/J is less favored for the induction of colitis because this strain is characterized by the possibility of developing a number of autoimmune diseases initiated by the Th1 phenotype [33]. This strain also has a high mortality rate, and it is difficult to keep two or more SJL/J mice together because of their aggressive nature [34].

The cellular and immune responses, as well as the secretion profile of oxazolone-induced colitis cytokines, differ from those of TNBS colitis. Oxazolone colitis is characterized by an intense production of IL-13 secreted by natural killer T-cells (NKT) of lamina propria CD4, instead of conventional CD4 + T-cells producing IFN-g. Above all, the blocking of IL-13 by a fusion protein IL-13Ra2-Fc prevented colitis, based on these data, and it can be very well understood that the IL-13 and NKT cells are involved in colitis to oxazolone, but this model failed to produce colitis in mice deficient in NKT cells [35]. These observations can be supported by data showing that NKT cells are cytotoxic for epithelial cells carrying NKT cell targets, and this cytotoxicity can be increased by IL-13 [35]. However, IL-13 has reduced the potential for epithelial barrier function by increasing apoptosis of epithelial cells, in addition to the permeability of tight junctions *via* claudin-2 and tricellulin [36]. This unfavorable effect of IL-13 on the epithelial barrier function is reinforced by IL-9 produced by Th2 cells [37].

### 2.4. Chemically Induced Model Limitations

The use of a chemically induced IBD model requires the study of several variables to require into consideration. Always work with the same protocols to ensure that the studies are reproducible; you ought to closely monitor the batch of chemicals, strain, gender, animal source, chemical supplier, dosage level, frequency, and duration. This sort of model can also be severe; the TNBS presents an accentuating gravity compared to the DSS models [38].

## 3. Adoptive T-Cell Transfer Experimental Model

**Figure 1:**
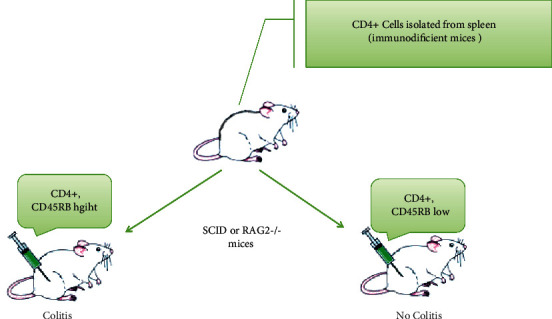
Colitis induction in SCID mice. Experimentally, the adoptive T-cell transfer model induces chronic small bowel and colonic inflammation, which resembles some key aspects of human IBD. To generate the model, CD4^+^CD45RB^high^ T-cells (which are CD25^–^) are sorted and isolated from donor BALB/c splenocytes. Cell transfer to a syngeneic immunodeficient SCID or RAG2^–/–^ recipient generates a model with primary inflammation in the colon.

A significant progress in the development of mouse models of intestinal inflammation shows that the adoptive transfer of naive CD4^+^ T-cells (CD4^+^CD45RB^high^ T-cells) from donor mice to SCID or RAG1 mice/syngeneic immunodeficient (lymphopenic) SCID or RAG1 mice/recipients induces an aggravation of the disease and a specifically colonic inflammation, which develops 5 to 10 weeks after the treatment. On the other hand, the transfer of mature CD4^+^CD45RB^low^ T-cells or the cotransfer of naive and mature T-cells to recipient mice failed to cause colitis (Figure 1) [39, 40].

We start from the assumption that T-lymphocytes have function differences, which express the various CD45 isoforms; we studied the expression of CD45RA, CD45RO, and CD45RB by peripheral and intestinal T-lymphocytes of IBD patients and compared them to healthy patients. In order to test the functional difference between CD45RB^high^ and CD45RB^low^ cells, peripheral and intestinal-derived T-lymphocytes were sorted and stimulated with CD3/CD28. The pro- and anti-inflammatory cytokine profiles of these cells have been determined [41].

The colitis of the CD45RB model is mediated by Th1 responses associated with IFN-g and TNF-*α* productions [8]. TNF-*α* is derived primarily by non-lymphoid cells in the recipient [42]. IL-6 trans-signaling is required for this colitis [43], and animal models of IBD IL-4 may indirectly promote Th1-type immune responses to sustain the colitis [44]. IL-4 blocks the TGFb-dependent development of Foxp3^+^ Treg cells and induces a unique CD4^+^ T-cell population that plays a pathogenic role in the colitis through productions of both IL-9 and IL-10 [45]. In order to be colitogenic, differentiation/expansion of donor-derived naive T-cells is induced even in the absence of gut-associated lymphoid tissues such as Peyer's patches and mesenteric lymph nodes [46].

Several regulatory T-cell subsets have been identified using the CD45RB model. Colitis in this model is suppressed by TGFb1-producing memory CD4^+^ T-cells (Th3 T-cells) [47] and by an antigen OVA-specific IL-10-producing memory T-cell subset termed Th1 [48]. More significantly, various studies regarding CD4 CD25 Foxp3 Treg were performed using this model. One of the important questions in IBD is: although Treg is accumulated in the inflamed colon of IBD patients, why is it unable to effectively suppress the inflammation presented? The CD45RB model can provide an answer to this question. One possibility is that Treg function may be impaired in patients with IBD. This possibility is confirmed by the fact that Treg cells are unable to keep up their Foxp3 expression and their regulatory activity within the absence of paracrine IL-10 [49].

Indeed, the absence of IL-10-inducing STAT3 signaling in Treg cells abolishes their ability to inhibit Th17 and IFN-g + Th17 cell responses and colitis development in the CD45RB model [50, 51]. Another possibility is that effector T-cells cannot respond to Treg cells in IBD patients. This possibility is supported by that cotransfer of naive CD4^+^ T-cells from Smad7 Tg mice with WT Treg cells induces colitis in recipient RAG1 KO mice; on the contrary, colitis is not observed when naive CD4^+^ T-cells from WT mice were cotransferred with WT Treg cells [52].

In particular, there is a specific CD4 T-cell population, which expresses both Foxp3 and IL-17, in the inflamed colon of CD, but not in patients with UC [53]. In addition, CD4^+^ Foxp3^+^ IL-17^+^ T-cells develop in the colon of the CD45RB model.

The IL-23 receptor is a special gene for sensitivity to IBD; polymorphisms are negatively related to the development of IBD. However, the pathogenic role of IL-23 and its receptor has been well demonstrated in the CD45RB model [54, 55].

### 3.1. Adoptive T-Cell Transfer Model Limitation

Considering that the researchers use immunodeficient mice to develop this model, you should know that a complete overview of the generation of colitis is not possible [38].

## 4. Genetically Engineered Experimental Model

### 4.1. IL-10 Knockout Model

The development of murine models used to study the pathophysiology of IBD has been maintained after the invention of a model with a genetic aspect, using mice that have an IL-10 deficiency [56]. This model allowed giving great importance to the anti-inflammatory function of IL-10; on the other hand, it turned out that the genetic polymorphisms brought to the IL-10 locus, led to the development of Crohn's disease and ulcerative colitis [57, 58]. In addition, there is a familial form of Crohn's disease that has been identified and that is due to a homozygous mutation in the IL-10 receptor subunits: IL-10R and IL-10RB [59].

IL-10 is a well-known regulatory cytokine and represents a key IBD (both UC and CD) susceptibility gene. IL-10 KO mice, which are genetically engineered to lack the IL-10 gene, spontaneously develop colitis after 3 months of age [14, 15].

Mice with targeted deletion of IL-10 (Il10/) develop spontaneous inflammation of the colon characterized by the presence of an inflammatory infiltrate made up of lymphocytes, macrophages, and neutrophils [56]. The inflammation is initially driven by a pro-inflammatory Th1 T-cell response and as such is ameliorated by systemic administration of anti-IL-12p40 and to a lesser extent anti-IFN-g [60]. However, for as yet unexplained reasons, the production of IL-12 and IFN-g decreases over time and is to some extent superseded by progressive increases in the production of the Th2 cytokines IL-4 and IL-13 [61]. Blimp1/and Il10rb/mice, which also have a defect in IL-10 production or responsiveness, recapitulate the phenotype of Il10/mice and thus support the presumption that lack of IL-10 production is directly responsible for the intestinal inflammation in such mice [62]. In addition, deletion of IL-10 in all T-cells or specifically in Foxp3^+^ Treg cells also results in spontaneous colitis, indicating that IL-10 derived from these cells is important to the maintenance of gut homeostasis [63].

### 4.2. IL-10^–/–^ Knockout Mouse Limitations

One limitation of this model is that substantial variability in colitis development can occur between facilities. This is due to the model being highly dependent on microbiome differences. There is also the long disease development time, though colitis onset can be accelerated and synchronized by using piroxicam. This itself needs to be carefully validated with regards to dose, formulation, and the age and microbial status of the mice. There is also no weight loss observed in this model. You can monitor other clinical indicators instead, but some of them are only seen with severe disease. A more efficient, sensitive, and validated method to monitor inflammation levels is to measure lipocalin 2 in the feces [38].

## 5. Spontaneous Mutation Mouse Models

### 5.1. SAMP1/YitFc Colitis

The SAMP1/YitFc mouse strain represents a model of Crohn's disease (CD) like ileitis that is ideal for investigating the pathogenesis of chronic intestinal inflammation. Variously from the vast majority of animal models of colitis, the ileal-specific phenotype characteristic of SAMP1/YitFc mice occurs spontaneously, without genetic, chemical, or immunological manipulation. In addition, SAMP1/YitFc mice possess remarkable similarities to the human condition in regard to disease location, histologic features, incidence of extraintestinal manifestations, and response to conventional therapies [64].

The discovery and characterization of a vast number of animal models of intestinal inflammation have greatly advanced our understanding of the pathogenesis of IBD [65]. These models have provided extensive data to support the general hypothesis that IBD results from a dysregulated immune response, triggered by environmental factors in a genetically susceptible host [66]. Innate mice provide a unique opportunity to study the influence of predisposing genes and environmental factors in the pathogenesis of IBD. The SAMP1/Yit murine model, initially described by Matsumoto et al. [67], was developed by brother-sister mating of SAMP1 mice displaying skin lesions, which correlated closely with the presence of intestinal inflammation. Although the precise cause of ileitis in these mice is unknown, it is likely that genetic as well as environmental factors, such as the indigenous bacterial flora, play an important role [68]. SAMP1/Yit mice spontaneously develop lesions in their terminal ilea, reminiscent of those originally described in humans by Crohn et al. [69]. Several features of the SAMP1/Yit model make it very attractive to study the mechanisms of chronic intestinal inflammation: (1) intestinal inflammation develops spontaneously without chemical, immunologic, or genetic manipulation; (2) lesions are located primarily in the terminal ileum; (3) the terminal ileum displays a discontinuous pattern of inflammation, with areas of normal mucosa alternating with areas of transmural involvement; (4) chronic ileitis occurs in virtually all animals; and (5) 30- to 40-week-old mice display a severe, chronic ileitis that does not resolve spontaneously [67, 69]. A colony of SAMP1/Yit mice was developed at the University of Virginia from 2 breeding pairs obtained from Japan in 1996. After more than 20 generations of continuous brother-sister mating, several phenotypic changes have appeared within the colony [70].

### 5.2. Derivation and Histopathology of the SAMP1/YitFc Strain

Originally, a colony of AKR/J mice was developed at Kyoto University in Japan from several pairs of founder mice purchased from The Jackson Laboratory (Bar Harbor, MN); SAMP1/YitFc mice represent a substrain of AKR/J mice was produced through a program of selective breeding. After a number of generations of brother-sister breeding, litters of mice were produced that spontaneously expressed an accelerated senescent phenotype, likely due to an accidental outcross with a non-AKR strain, and were referred to as senescence-accelerated mice (SAM) [71].

In 1975, the propagation of ten lines of “senescence-prone” mice SAMP1^–^ [72] resulted from brother-sister mating with a selection of breeders with accelerated senescence, shortened life span, and other signs of pathology. Among the clinical phenotypes noted in these senescence-prone mice were severe skin lesions and other autoimmune manifestations [73]. A SAMP1/Yit substrain was subsequently generated from the SAMP1 line by selective breeding of littermates that exhibited skin lesions and ileitis. By the 20th generation of brother-sister mating, this substrain had lost the premature senescent phenotype but consistently developed terminal ileitis [68].

Dr. Satoshi Matsumoto at the Yakult Central Institut of Microbiological Research in Tokyo carried out the initial development of the SAMP1/Yit mouse [68]. These mice were described to develop both acute and chronic intestinal inflammation that primarily localized to the ileum and cecum with a discontinuous pattern. The inflammatory infiltrate consisted of lymphocytes, macrophages, and neutrophils, with crypt microabscesses in older mice, and was accompanied by progressive disruption of the epithelium, tissue atrophy, and crypt elongation [68].

### 5.3. The SAMP1/Yit Mouse as a Model for Crohn's Disease

Based on a previous study of Kosiewicz et al. who have clearly demonstrated the SAMP1/Yit mouse models [74]. The mucosal inflammation, first described by Matsumoto and his colleagues [68], centered in the small intestine, is marked by a discontinuous inflammation and consists of inflammation containing granulomata. These cardinal features of Crohn's disease are not shared with most previous models, indicating that the SAMP1/Yit mouse is a singular model of the human disease and should yield fundamental insights into its immunopathogenesis. Additionally, since the disease in the SAMP1/Yit mouse is localized to the small intestine, it is likely that the resident organisms in this part of the bowel produce the antigens that cause both the SAMP/Yit phenotype and Crohn's disease [75].

The current work also extends our knowledge of the mucosal inflammation in the SAMP1/Yit model in several respects. First, as is seen in Crohn's disease, T-cells present in the mouse lesions produce IFN-*γ*. Moreover, these cells can be used to transfer disease to normal recipient mice, suggesting that they are actually the basis of the inflammation in this model. Finally, the authors display, using this adoptive transfer procedure, that disease can largely be prevented by administering anti-TNF-*α*. The success of this therapy marks yet another significant parallel with human Crohn's disease and raises the hope that other agents that are found to ameliorate the SAMP1/Yit phenotype will be of use in treating Crohn's disease [75].

While the data so far accumulated on the SAMP1/Yit mouse already occurs important insights into the nature of this disease of mice, important questions remain to be addressed as they have been, to varying extents, in other models. Is the Th1 disease that occurs spontaneously in SAMP1/Yit mice the product of an excessive Th1 response, as in the case of STAT4 transgenic mice, or is it caused by an inadequate counter-regulation, as in the SCID transfer model? Which organisms in the microflora contribute most to the Th1 response? Are some organisms protective, as has been seen in IL-10-deficient mice? Finally, what are the genetic factors that lead to this remarkable phenotype? SAMP1/Yit mice can be interbred with mice of different genetic backgrounds in order to map and, eventually, isolate the relevant genes involved in SAMP1/Yit colitis. Given the remarkable similarity between this condition and the human disease, the answers to these questions may well apply directly to Crohn's disease [75].

### 5.4. Spontaneous Mutation Model Limitations

One major limitation of spontaneous IBD models is the time needed for full disease penetrance. For example, for the SAMP1/Yit substrain, 100% penetrance takes approximately 30 weeks. This can result in long and costly study timelines [38].

## 6. Microbiome-Induced Mouse Models

### 6.1. The Gut Microbiota in IBD

The human gut includes 100 trillion different microbial organisms, containing bacteria, viruses, fungi, and protozoa, which constitute the microbiota (also referred to as the microbial flora) [76]. Based on culture-independent molecular methods, more than 1,000 species of bacteria reside in the gastrointestinal tract and the collective genome of intestinal microbes is estimated to contain approximately 100 times more genes than the human genome [77].

**Figure 2:**
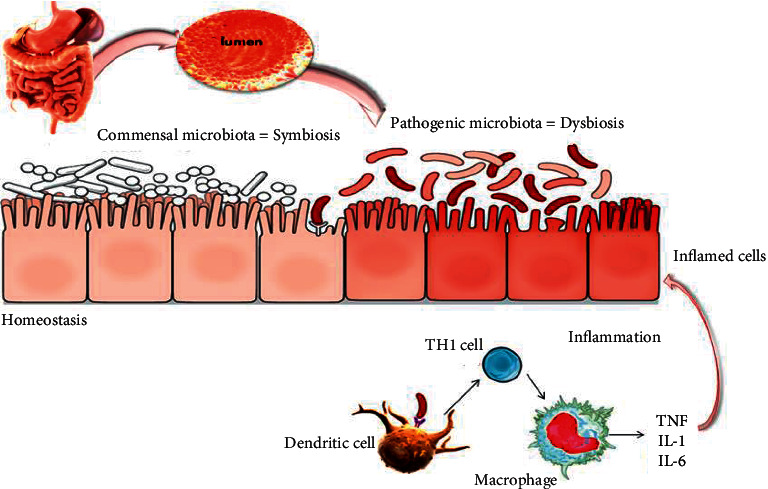
Mechanisms of inflammatory bowel disease. The intestine shelters a large diversity of microbiota that are in perfect balance (symbiosis); sometimes, this balance can be affected by many factors leading to the appearance of pathogenic bacteria that can alter the intestinal barrier and lead to the development of inflammatory bowel disease. The stimulation of the mucosal immune system may occur as a result of the penetration of bacterial products through the mucosal barrier, leading to their direct interaction with immune cells, especially dendritic cells and lymphocyte populations, to promote a classic adaptive immune response. Alternatively, bacterial products may stimulate the surface epithelium, possibly through receptors that are components of the innate immune-response system; the epithelium can, in turn, produce cytokines and chemokines that recruit and activate mucosal immune cells. Activation of classic antigen-presenting cells, such as dendritic cells, or direct stimulation through pattern-recognition receptors promotes the differentiation of type 1 helper T-cells (Th1) in patients with Crohn's disease (shown here) or, possibly, atypical type 2 helper T-cells in patients with ulcerative colitis. The stereotypical products of Th1 promote a self-sustaining cycle of activation with macrophages. In addition to producing the key cytokines that stimulate Th1 (interleukin-12, interleukin-18, and macrophage migration inhibitor factor), macrophages produce a mix of inflammatory cytokines, including interleukin-1, interleukin-6, and most notably tumor necrosis factor, which target a broad variety of other types of cells. The latter include endothelial cells, which then facilitate the recruitment of leukocytes to the mucosa from the vascular space. Most important, these functions may be altered either by genetically determined variants, as exemplified by germ-line mutations in the gene encoding NOD2, the product of the IBD1 locus, in some patients with Crohn's disease, or by environmental factors.

In the case of dysbiosis observed by a lack of balance between the various commensal bacteria of the intestine, a weakening of mucosal defenses promotes intestinal epithelium permeabilization resulting in more frequent contacts between the commensal flora and the mucosal immune system. In CD, these interactions would still be facilitated through a lack of bacterial clearance by macrophages in which secretion of inflammatory cytokines is defective [78]. An excess of such interactions would cause a loss of tolerance to commensal flora by activating mucosal dendritic cells and the sentinel cells of innate immunity (Figure 2).

The anti-inflammatory capacities to decrease the number of bacteria and the increase of bacteria with inflammatory capacities are observed in patients with IBD when compared to healthy individuals [79]. The most consistent changes are a reduction in the diversity of gut microbiota and the lower abundance of Firmicutes [77, 79–81]. Increases in abundance of Proteobacteria and Bacteroidetes have been reported, but reductions have also been reported [79, 81]. *Faecalibacterium prausnitzii*, which belongs to Clostridium cluster IV, has been reported to have an anti-inflammatory effect by producing butyrate. It has been demonstrated that *F. prausnitzii*, *Blautia faecis*, *Roseburia inulinivorans*, *Ruminococcus torques*, and *Clostridium lavalense* are decreased in patients with CD when compared to healthy subjects [82, 83] and that the number of *F. prausnitzii* is correlated with the risk of relapse of ileal CD after surgery. The defect of colonization of *F. prausnitzii* was observed in UC patients during remission and the recovery of the *F. prausnitzii* population after relapse is associated with the maintenance of clinical remission [84]. In addition, Sokol et al. showed that human peripheral blood mononuclear cells stimulated with *F. prausnitzii* induce the production of IL-10 and inhibit the production of inflammatory cytokines, such as IL-12 and IFN-*γ* 18936492. Furthermore, a significant decrease of *Roseburia* spp. was shown in the gut microbiota of healthy individuals with a high genetic risk for IBD.

In IBD patients, the disruption of the gut microbiota affects the production of metabolites. For notable example, the concentration of SCFA has been shown to typically decrease in IBD patients due to butyrate-producing bacteria, such as *F. prausnizzi* and Clostridium clusters IV, XIVa, and XVIII [83]. In fact, the decrease in SCFA production affects the differentiation and expansion of immune Treg cells and the growth of epithelial cells, which occupy a considerable role in maintaining intestinal homeostasis. Moreover, in patients with IBD the number of sulfate-reducing bacteria, such as *Desulfovibrio*, is higher than in healthy patients [85, 86], causing the production of hydrogen sulfate that damages the cells of the epithelium intestinal and induces the development of mucosal inflammation [85, 87]. Commonly, these data significantly show that alteration of the gut microbiota is associated with the pathogenesis of IBD.

### 6.2. The Non-Bacterial Microbiota and IBD

Currently, most of the studies on the link between inflammation and the microbiota have focused on bacteria. Nevertheless, the microbiome also contains fungi and viruses, and the role of these microorganisms in the various functions of the organism, as well as in the development of certain diseases, is more and more valued. Metagenomic sequencing analyzes of viral particles isolated from fecal samples have shown that the intestinal virome is exceptionally composed of bacteriophages [88–90]. Because of metagenomic techniques, they were able to detect changes in the composition of bacteriophages associated with IBD, especially an increase in the sequences of Caudovirales bacteriophages in ileal biopsy samples and intestinal washing of pediatric patients with Crohn's disease [91, 92]. In notable addition, the expansion of Caudovirales bacteriophages is associated with a reduction in bacterial diversity. After all of these studies, direct questions should be proposed about the direct role in the pathogenesis of IBD or whether they simply reflect an underlying dysbiosis. Likewise, a distinct role for eukaryotic viruses in IBD has not been established [93].

### 6.3. Mouse Models of Bacterium-Induced Inflammation

Mice were considered the most suitable model for examining the complex relationship of bacteria and specific bacterial factors to the pathogenesis of IIDs. Especially, studies that have been done on sterilized mice without germs, without specific pathogens (sPF), and gnotobiotics reimplanted by a known microbial flora have shown that bacteria play an essential role in the initiation and development of chronic intestinal diseases. A crucial discovery has shown that non-germinated IL10^–/–^ mice that are not developed do not develop enterocolitis, conversely to the same mice maintained under sPF conditions inoculated with an sPF flora after gnotobiotic birth [94]. In this study, the characteristics of the disease also differ depending on the age at which the mice were inoculated with the respective microbiota. These results have shown for the first time that bacteria are certainly important for the initiation of intestinal inflammatory conditions such as IBD, even in the visible presence of an immune predisposition. From that time, several research groups, using mouse models infected with specific bacteria or a complex microbiota, have focused on the question of which specific bacteria or bacterial communities and which of their functions or individual factors could be intimately associated with symptoms IBD type [95].

Table 1 summarizes experimental models of inflammatory bowel disease.

### 6.4. Limitations of Current IBD Mouse Models

- Most mouse models typically depend on gene knockouts, while human alleles rarely cause complete loss of function
- Mouse models typically study the direct effect of a single gene, while in humans, there are often multiple alleles involved
- The presence of a large discrepancy between mice and humans with regard to immune responses
- Mice do not correlate to genetic and environmental diversity in human populations
- Experiments on mice do not consider dependent variables such as drugs, smoking, and food inherent in human research [96]

## 7. Conclusion

IBD mouse models remain a crucial tool for preclinical research and drug development; each model undoubtedly has specific advantages/limitations over other models, such as the CD45RB model which provided useful information to understand the adaptive immune system mechanisms associated with the pathogenesis of IBD. By comprehending, the fundamental differences through the available range of IBD models researchers can select the most appropriate mouse models to use on a study-by-study basis.

## Figures and Tables

**Table 1:** Summarized table of experimental models of inflammatory bowel disease.

Classification	Types	Characteristics	References
Chemical model	DSS	Damage to epithelial cells and mucosal barrier function	Chassinng et al. [16]
		Erosions/ulcers, loss of crypts, and infiltration of granulocytes	Okayasu et al. [17]
	TNBS	Immune response mediated by Th1 and characterized by an infiltration of CD4 T-cells, neutrophils, and macrophage	da Sliva et al. [20]
		Leading to transmural colitis	Martinez-Moya et al. [22]
	Oxazolone	Immune response mediated by Th2 cells	Kojima et al. [31]
		Inflammation of the mucous membranes, epithelial microulcerations, and histopathological changes in the distal colon	
Immunological model	Adoptive T-cell transfer	Immune response mediated by Th1 cell linked to the production of IFN-g and TNF-a	Corazza et al. [42]
Genetic model	IL-10 knockout	Inflammatory response mediated by Th1 cells	Berg et al. [60]
Spontaneous mutation model	Samp1/YitFc	Intermittent inflammation and consists of inflammation containing granulomas	Kosiewicz et al. [74]
Microbiome-induced models	Gut microbiota	The diversity of the intestinal microbiota decreases and is enriched with Firmicutes; there are also increases in the number of proteobacteria and bacteroids	Qin et al. [77] and Frank et al. [79]
			Manichanh et al. [81]
